# Hand therapy or not following collagenase treatment for Dupuytren’s contracture? Protocol for a randomised controlled trial

**DOI:** 10.1186/s12891-019-2712-z

**Published:** 2019-08-28

**Authors:** Terese Aglen, Karin Hoegh Matre, Cecilie Lind, Ruud W. Selles, Jörg Aßmus, Tina Taule

**Affiliations:** 1 Department of Occupational Therapy, Orthopaedic Clinic, Haukeland University Hospital (HUH), Bergen, Norway; 2 Department for Plastic-, Hand-, Reconstructive surgery and National burn unit, Surgery Clinic, Haukeland University Hospital (HUH), Bergen, Norway; 3 Department of Rehabilitation Medicine and department of Plastic and Reconstructive Surgery Hand Surgery, Erasmus MC – University Medical Center, Rotterdam, The Netherlands; 4 Centre for Clinical Research, HUH, Bergen, Norway

**Keywords:** Occupational therapy, Physical therapy, ADL, Activity, COPM, URAM, ROM, Hand exercise, Splint, Orthosis

## Abstract

**Background:**

Dupuytren’s contracture (DC) is a fibrotic hand condition in which one or more fingers develop progressive flexion deformities. Quality of life is diminished due to disabling limitations in performing everyday activities. For DC patients treated with collagenase, referral for subsequent hand therapy is inconsistent. It is unknown whether subsequent hand therapy is beneficial compared to no therapy. The purpose of this study is to determine whether hand therapy improves DC patients’ performance of and satisfaction with performing everyday activities one year after collagenase treatment.

**Methods:**

We will conduct a randomised controlled trial with two treatment groups (hand therapy vs. control) of DC patients who have received collagenase treatment. DC patients with contracted metacarpophalangeal joint(s) (MCPJ) (hand therapy, *n* = 40; control, n = 40) and those with proximal interphalangeal joint(s) (PIPJ) involvement (hand therapy, n = 40; control, n = 40) comprise two subgroups, and we will study if the treatment effect will be different between both groups (*n* = 160). Patients with a previous injury or treatment for DC in the treatment finger are excluded. Hand therapy includes oedema and scar management, splinting, movement exercises, and practice of everyday activities. The main outcome variable is patients’ performance of and satisfaction with performing everyday activities, as assessed with the Canadian Occupational Performance Measure. Secondary outcomes are DC-specific activity problems, as assessed with the Unité Rhumatologique des Affections de la Main scale, and active/passive flexion/extension of treated joints and grip force using standard measuring tools, and self-reported pain level. Demographic and clinical variables, degree of scarring, cold hypersensitivity, number of occupational sick-leave days are collected. Self-reported global impression of change will be used to assess patient satisfaction with change in hand function. Assessments are done pre-injection and 6 weeks, 4 months, and 1 year later. Standard univariate and multivariate statistical analyses will be used to evaluate group differences.

**Discussion:**

This study aims to assess whether hand therapy is beneficial for activity-related, biomechanical, and clinical outcomes in DC patients after collagenase treatment. The results will provide an objective basis for determining whether hand therapy should be conducted after collagenase treatment.

**Trial registration:**

This study has been registered at ClinicalTrials.gov as NCT03580213 (April 5, 2018).

## Background

Dupuytren’s contracture (DC), also known as Dupuytren’s disease, is a progressive fibroproliferative disorder affecting the palmar and digital fascia of the hand [1]. The development of nodules and cords in the palm may lead to flexion deformity and limitation of function [2, 3]. DC is autosomal dominant with variable penetrance. Prevalence estimates depend on geographic location, mail gender and increase with age [1]. In Sweden 56% of the diagnosed DC patients received treatment [4]. DC affects both performance of everyday activities and quality of life [5, 6]. The most disabling self-reported limitations in activity include difficulties in body washing and grooming, putting on gloves, shaking hands, and doing carpentry [6, 7].

Currently only symptomatic treatment exists for DC patients [8]. The only nonsurgical treatment approved in Europe is direct injection of collagenase clostridium histolytic (CCH) into a pathological cord [9]. CCH enzymatically dissolves collagen, allowing the contracted cords to be disrupted and eased by gentle force 1–2 days after injection [10]. CCH is an effective and safe treatment for DC, as shown in a five-year follow-up study [11]. CCH is considered to be successful when a treated joint can be extended to 0–5° of full extension and clinical improvement exceeds 50% reduction of the contracture [12]. Patients treated in our clinic with CCH for DC are treated with one injection in one to three fingers. If the skin is tight during the extension procedure two days later, a few also get a needle fasciotomy performed in the same procedure. It is up to the surgeon if the patient is referred for hand therapy or not within 1–2 weeks.

Inflammatory responses resulting from collagenases’ mechanism of action are reported, in addition to some complications; oedema, local contusions, scarring, haematoma, persistent joint contracture, pain, poor finger flexion, reduced grip strength, skin lesions, and tendon lesions [2, 13–17]. Patient satisfaction deteriorates with time if the contracture recurs [18, 19].

DC patients’ positive view of hand function after treatment is based on self-evaluation of whether their previous activity limitations have diminished [20]. It seems reasonable to hypothesize that hand therapy after CCH treatment might help to improve performance of these activities and temper other DC-related issues.

Hand therapy plays an important role in helping patients regain maximum function and minimizes further disability [13]. It is a symptom-based and clinically reasoned treatment that depends on an individual patient’s outcomes and circumstances [21, 22]. Recommendations for hand therapy after treatment for DC vary in the literature and in practice. Patient education, oedema management [23–25], wound care and scar management [26–28], splinting, exercises, passive stretching, and graded return to everyday activities are mentioned [13].

To our knowledge, the only study evaluating a post-CCH hand therapy protocol alone, relates to patients having a severely contracted proximal interphalangeal joint (PIPJ) [29]. In this case, hand function improved after 4 weeks of hand therapy following CCH treatment. However, lacking a control group, this protocol needs further evaluation, especially using long-term follow-up. In two other studies mentioning post-CCH treatment, DC patients wore a night splint, and in one study, patients also performed exercises [12, 30].

The primary goal of splinting for DC is to reduce the risk of recurrence and to prevent flexion contracture caused by scarring [31]. If the CCH treatment is unsuccessful, a splint might promote more joint extension [32, 33]. Targeted splinting for PIPJ contracture has produced good results after 4 weeks of splinting [29]. However, four systematic reviews all conclude that evidence in the literature does not support night splinting for DC post-surgery [21, 31, 34, 35]. It is clear from these studies, however, that compliance is important. No definitive conclusions can be made about the possibility that hand splinting might be beneficial post CCH. Reasons why patients discontinued using the splinting are many [22]. Optimal moulding of the splint, and the principle of applying low-grade force for longer periods of time, must be followed to produce tissue change without tissue micro-tears if the purpose of the splint is to straighten the joint further [32, 33].

With DC, ligaments and associated tissues have been in a shortened position for a period of time. To recover ROM, the patient’s finger ligaments and associated tissues needs to be elongated through finger exercise, use of the hand, and splinting. Exercises that maintain the metacarpophalangeal joint (MCPJ) in flexion, and at the same time, allow the PIPJ to be actively extended will improve PIPJ extension. In order to lengthen an oblique retinacular ligament that is frequently in a contracted or shortened condition, the PIPJ can be held in full extension, and the distal interphalangeal joint (DIPJ) can be exercised through active movement [32]. General hand exercises are necessary if the fingers have reduced function, as will general use of the hand in everyday activities.

In our literature search, no studies emerged that looked at the use of activities to improve function following CCH treatment. In a RCT on hand injury rehabilitation, patients with a hand injury improved more after an occupation-based intervention combined with exercises than when they performed exercises only [36]. Patients in the intervention group incorporated specific activities into their daily lives. Performing meaningful occupation-related tasks instead of rote exercises is critical for the efficacy of constraint-induced therapy following median and ulnar nerve injuries [37]. Overall hand function improved more when patients performed purposeful activities that mimicked typical everyday activities than when they performed exercises alone [38]. Performing purposeful activities accelerates the recovery of hand injuries, it also naturally increases the number of activity repetitions compared to engaging in non-purposeful activities [36]. Encouraging patients to use their affected hand more and more in everyday activities will also probably improve their actual performance of these activities and satisfaction with them.

In summary, no studies have compared the effects of hand therapy versus no therapy. Information about the effect of hand therapy post CCH is limited. As with other treatments, the body reacts to CCH treatment by invoking a healing process. Also, patients seek to improve activity performance. In this study we want to investigate if hand therapy amplify the healing process to prevent re-contracture and improve the patients’ performance of and satisfaction with everyday activities.

## Methods/design

### Aims

The main purpose of this study is to determine whether hand therapy administered following CCH treatment for DC improves patients’ performance of and satisfaction with performance of their everyday activities 1 year after CCH treatment. The comparison group will not receive hand therapy following CCH treatment for DC. In addition, this intervention will be evaluated in cases involving PIPJ contractions versus cases involving only MCPJ contractions.

### Hypotheses

- H1. After CCH treatment for DC, patients who receive hand therapy will improve their performance of everyday activities more than those patients who do not receive hand therapy.
- H2. After CCH treatment for DC, patients who receive hand therapy will be more satisfied with their performance of everyday activities than those patients who do not receive hand therapy.
- H3. Involvement of the PIPJ in the DC is a better predictor for improvement after receiving hand therapy than involvement of only the MCPJ.
- H4**.** The treatment effect (hand therapy vs. control) for the COPM will be greater for patients where PIPJ is involved than for that where MCPJ solely is involved

### Design

**Fig. 1:**
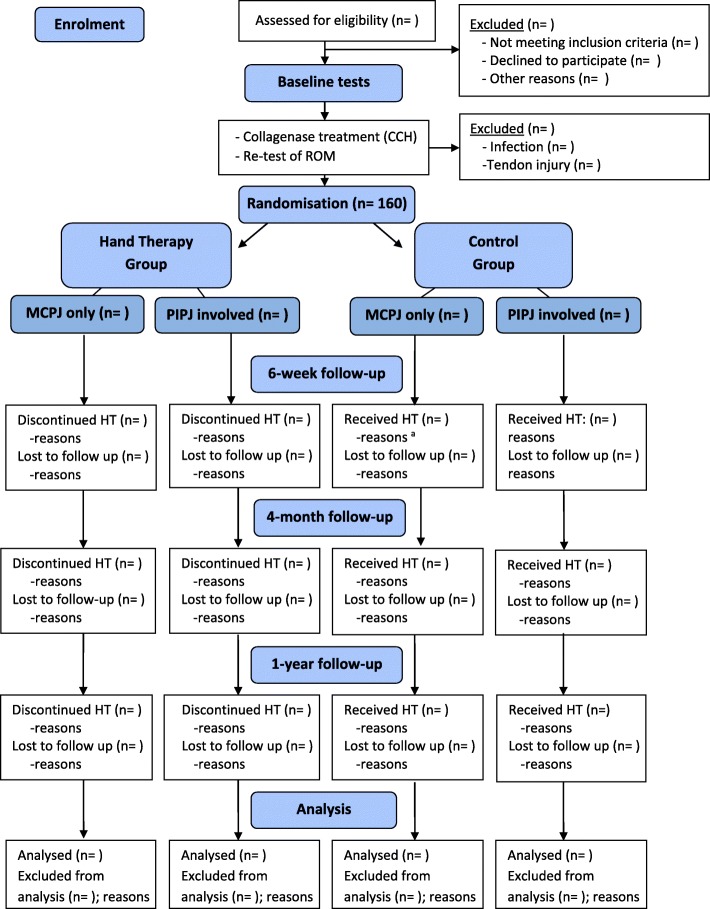
Flow chart illustrating the selection, allocation, intervention, and assessment schedule of patients with Dupuytren’s contracture**.** ^a^ Reasons for receiving hand therapy after allocation to the control group are developing CRPS or an infection. This will be noted and considered in the analysis

The possible benefits of hand therapy on the participants’ performance and satisfaction with performing their everyday activities will be investigated in addition to range of motion. A randomised controlled trial with two groups will be used (Fig. 1). One group will receive hand therapy after CCH treatment, and the other group will not. Participants will be randomly allocated into the two groups. Additionally, the two groups each have two equally sized subgroups: one with PIPJ involvement (MCPJ and PIPJ or PIPJ only), and the other with only MCPJ involvement. Thus, there will be four groups, (“MCPJ and PIPJ or PIPJ only” / “MCPJ only”) x group type (hand therapy/control) (Fig. 1). Differences on actual performance of activities and satisfaction with that performance, before and after hand therapy or not, will be evaluated among the four subgroups.

### Setting of the study

The Department of Occupational Therapy, Orthopaedic Clinic, Haukeland University Hospital (HUH) will oversee the study and will be ultimately responsible for conducting the study and results. Some participants may live far away from the treatment location, as the HUH serves a large geographical area around Bergen. The intervention will be carried out in an outpatient clinic at the hospital.

### Participants

This study is currently recruiting participants (study start date: 10th of April 2018). Adult men and women who have had their DC treated with one CCH injection in one-tree fingers with joints summarised to ≥30° extension deficit are eligible. Patients can participate only once. Reasons for exclusion are as follows:

- having previous treatment for DC in the same finger being considered for the intervention
- having previous surgery or a major injury that affects the same fingers movement
- having complex regional pain syndrome, infection, or an allergic reaction to the CCH before randomisation
- having a tendon or ligament rupture in the hands before randomisation
- a patient incapable of complying with a therapy program due to cognitive or language challenges.

Activity performance and satisfaction are affected by capacity of both hands. Therefore, participation in the study is appropriate only once. If any of the included participants receive DC treatment in the unparticipating hand, or for other fingers of the experimental hand, during the present trial, this will be noted and considered as an adjustment variable in the analysis. Previous injury or DC treatment would undoubtedly cause changes in the connective tissue and would likely confound the results of our study. Participants who develop complex regional pain syndrome (CRPS) or infection, diagnosed by a doctor, must undergo hand therapy regardless of whether it affects hand function severely. This will be considered in the analysis as an adjustment variable.

### Enrolment and assessment schedule

Prospective participants are identified from the patients on the waiting list for CCH treatment at our hospital. They are contacted by a postal letter in the post. In the letter they are invited to participate in the study in the same letter that informs them about the time schedule for CCH treatment. A written, informed consent form is signed by the patient before demographic and medical information are collected, and baseline testing is conducted, including ROM (see below). Immediately after the CCH treatment, ROM will be measured again. Then, participants will be randomly assigned to receive either hand therapy or no hand therapy. The intervention starts on the same day of the extension procedure (see below), just after group assignment. The first follow-up time for all outcome measures will be set at 6 weeks post-CCH, so that possible recurrence can be assessed at 1 year [39]. A second assessment time will occur at 4 months post-CCH, when the routine night-time splint is discontinued (see below). At one-year post-CCH, a final assessment will be done. This point was selected to allow time for the healing process to complete and to detect potential recurrences of DC [31, 39]. A flow diagram for selection and assessment of participants is shown in Fig. 1.

### Randomisation and blinding

Included participants will be allocated to the four groups by a block randomisation method, stratified for PIPJ affection in one or more of the treated fingers, and MCPJ contractures (without PIPJ affection in any of the treated fingers). The randomisation schedule will be prepared using software by an independent statistician not involved in the study. The project group will be blinded for the block size. The assignment to either the hand therapy group or the control group will be put in sealed opaque envelopes with numbers, following an order generated by the software. A list with the numbers of the envelopes will be marked when the envelopes are used. The list and envelopes will be stored in a private locked closet that is inaccessible to blinded study researchers. The envelope will be opened by the participant and a receptionist when the CCH treatment is finished. If the participant is allocated to the hand therapy group, the therapy starts at the day of the extension procedure. The surgeon is also blind to participant allocation, since the CCH treatment is finished before allocation. It is not possible for the therapist performing the hand therapy intervention to be blinded, but the person performing and managing the assessments will be blinded. The participant will be instructed not to inform the assessor of their allocation group, nor to reveal any related aspects during any of the assessments. By nature of PIPJ and MCPJ DCs, it is impossible to blind this subgroup allocation.

### Intervention

#### All participants

Participants in the two groups are all treated with a collagenase injection (i.e., CCH) and extension procedure. A needle fasciotomy is done in addition in the same procedure for a few. These will be marked, and considered as an adjustment variable in the analysis. If the extension procedure causes a wound, it is dressed, and the patient is informed on how to prevent infection. An employer sick note is provided if needed. Further intervention depends on group assignment.

#### Control group

Participants in the control group are discharged from hospital without further interventions, not even a post-treatment appointment with the surgeon. The participants in this group are neither informed of what exercises to do, nor are they to be fitted for a splint or cast. If an infection is detected or CRPS diagnosed by the personal general practitioner or the surgeon, treatment accordingly (most often hand therapy) will be initiated. The Budapest criteria for CRPS will be used [40]. If not diagnosed when meeting at the 6 weeks or 4 months re-tests, the tester will contact a MD to do it. If CRPS develops, this participant will be marked as treated with a deviation from the protocol (Fig. 1). This marking will also be done if it is revealed that a participant has received some kind of hand therapy outside of the hospital.

#### Intervention group

Hand therapy comprises the following. To ensure the best possible treatment, we will give each participant an information leaflet and a diary for them to record specific notes about their therapy experience. The information discusses oedema and scar management, correct night-time splinting, hand exercises, and everyday activities to be done for the entire post-CCH and follow-up periods. Participants of the hand therapy group are instructed by an occupational therapist experienced in hand therapy for DC at the day of the extension procedure. An extension splint is moulded, and night-time splinting begins at this time. Instructions on how to do hand exercises are also given. Specifically, participants are asked to and instructed how to perform the daily activities listed in the Canadian Occupational Performance Measure (COPM). Depending on the patient’s needs and progress of oedema, scarring, proper splint adjustments, ROM, and mastering everyday activities, the timing and the amount of therapy sessions will vary individually. The participants will at least be seen at 4 and 6 weeks, but mostly they will also be seen at 1–2 weeks post CCH-treatment. The participants will be instructed in how to do exercises, use their hand in daily activities as exercise and treat their scar or oedema on their own. Use of the routine night-time splint will be discontinued at 4 months. Some participants might voluntarily choose to use it beyond the 4 months; this is noted. Therapy sessions might continue after 4 months, as it sometimes takes time to elongate structures surrounding the joints. If there is a persistent rest-contracture, this is noted. The therapist and participant end hand-therapy sessions when no improvement of joint extension is achieved for two sessions with 2 months apart, or if full ROM is achieved, whichever comes first. The intervention group is free to contact the study’s occupational therapist at any time during the trial.

#### Oedema and scar management

Oedema treatment is standard, and comprises rest and elevation of the hand in combination with movement of the whole arm and use of the affected hand in everyday activities. Compression achieved with an elastic self-adhesive bandage or with a oedema glove is recommended for 12 h per day for prolonged oedema (i.e., lasting more than 72 h) as long as it persists.

Every laceration leaves a scar. If present, scar treatment is standard, comprising application of pressure through a splint or paper tape, and sometimes includes silicone treatment [23]. If the scar is hard or hypertrophic, use of a splint together with a silicone sheet is recommended [26–28]. Scar and oedema treatment is recommended for 12 h per day for 2 months or more, if needed [41].

**Fig. 2:**
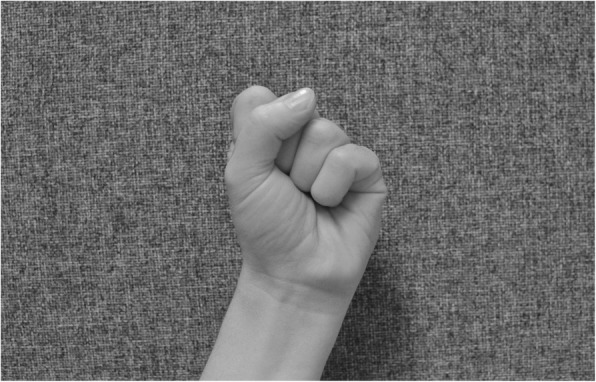
Make a fist

**Fig. 3:**
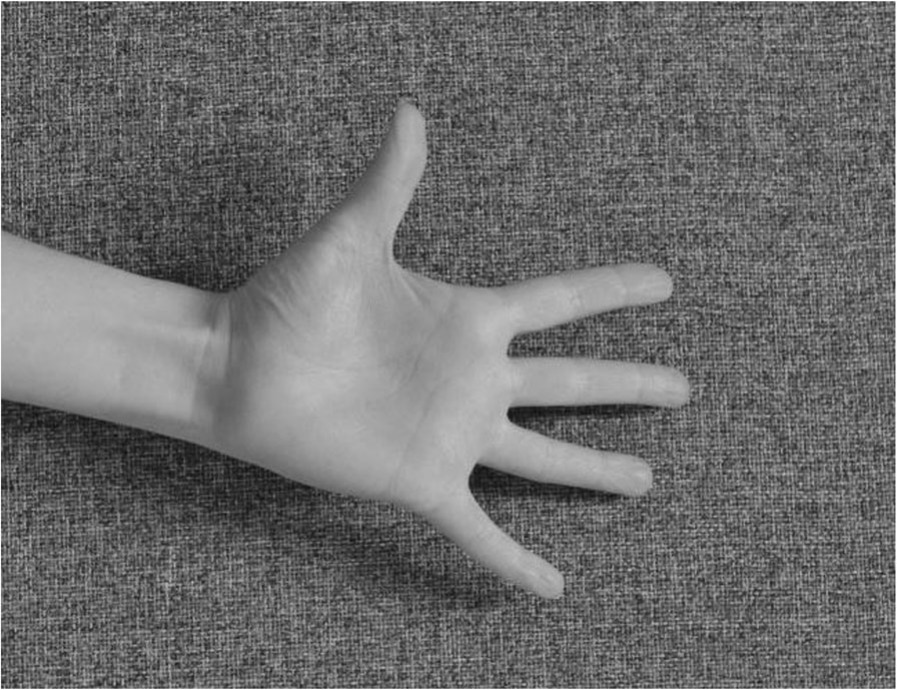
Stretch out the fingers

If needed for oedema management, exercises are performed as long as the oedema persists, and include the following:

- move arms up above the head and down again
- make a fist and stretch the fingers out (Figs. 2 and 3)
- keep the arm elevated by resting it on the chest when walking about or on a pillow for the first 2–3 days (inflammation-phase)

#### Splinting

**Fig. 4:**
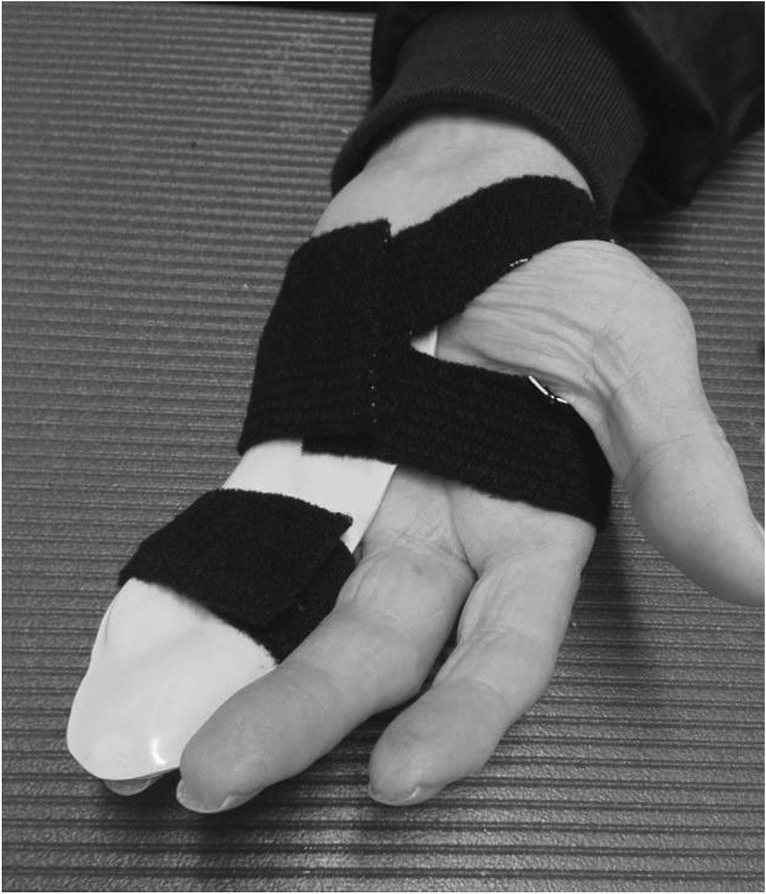
Night splint volar side

**Fig. 5:**
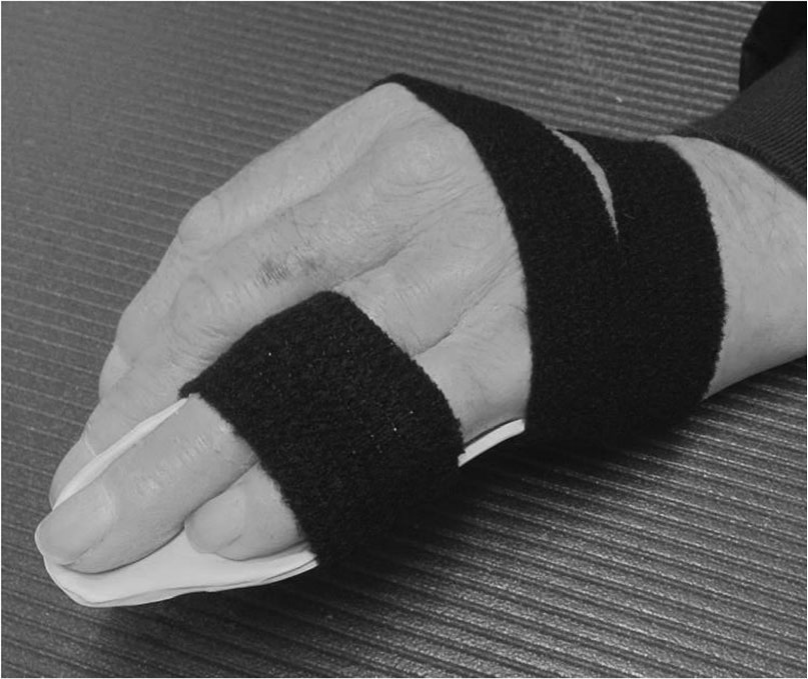
Night splint dorsal side

**Fig. 6:**
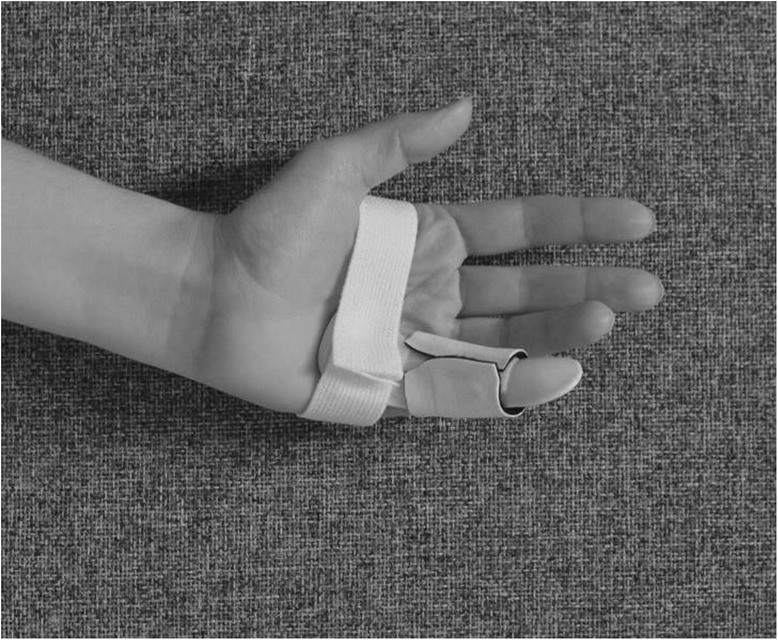
Finger gutter splint, volar side

**Fig. 7:**
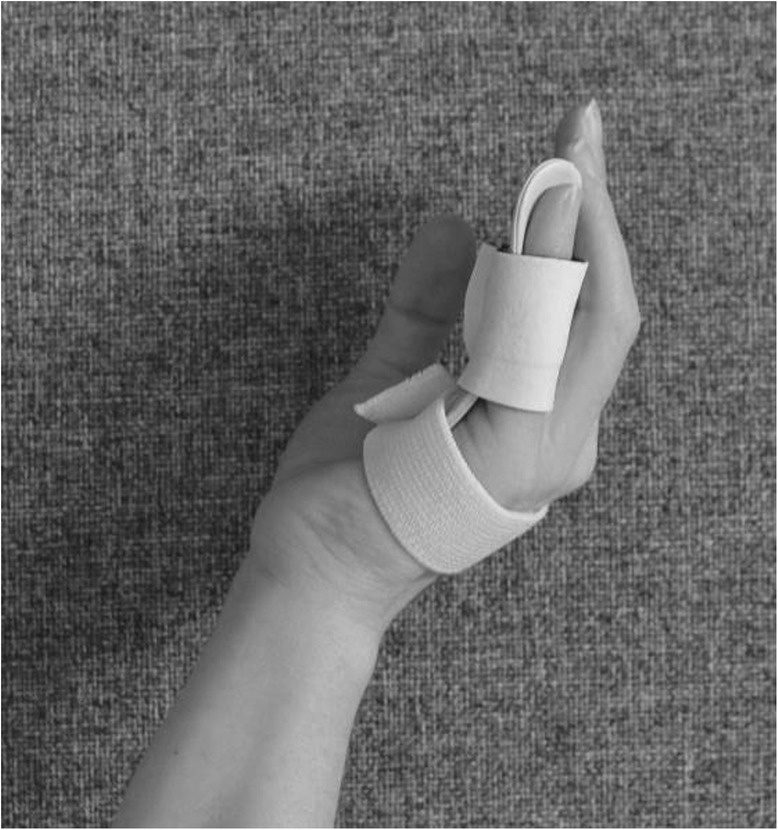
Finger gutter splint, dorsal side

**Fig. 8:**
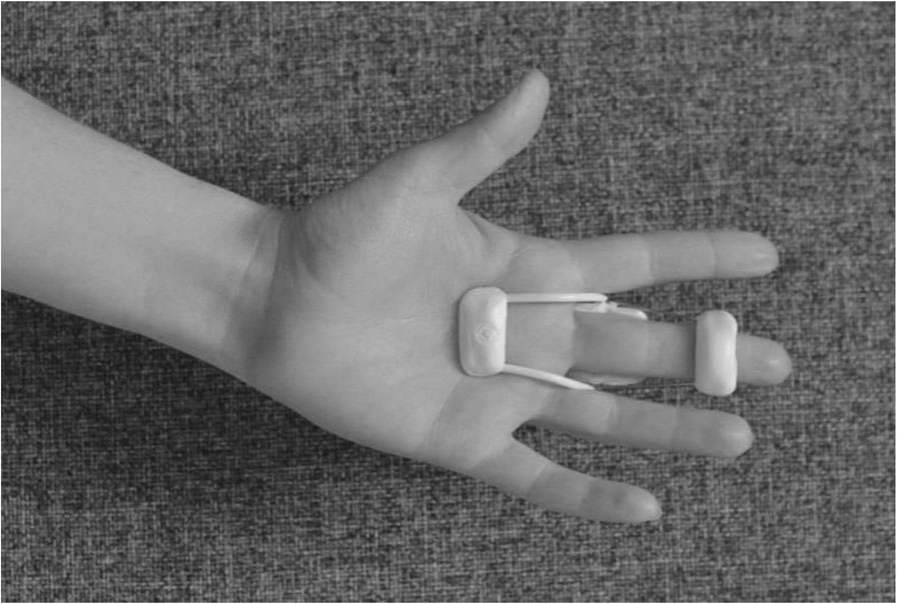
Finger three-point splint volar side

**Fig. 9:**
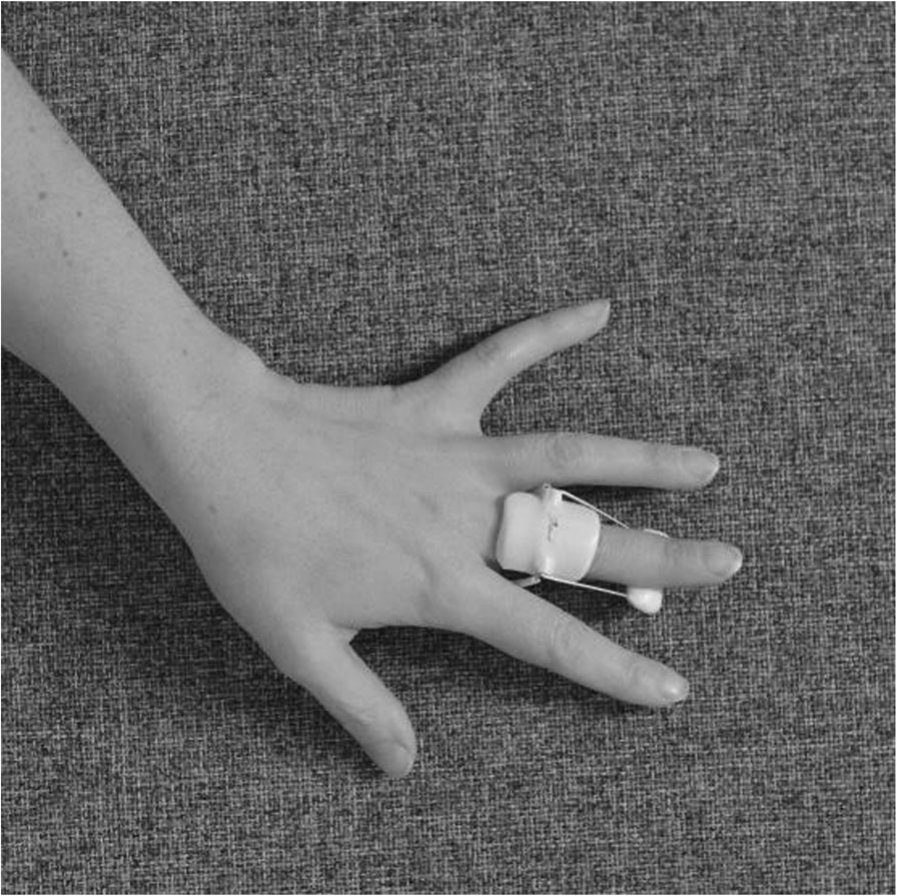
Finger three-point splint dorsal side

The splint is an individually moulded volar splint made of thermoplastic. It is custom-formed to accommodate the treated finger(s) and the neighbouring finger, with the MCPJs slightly bent and PIPJs extended (Figs. 4 and 5). As the hand is sedated at the time the splint is moulded, we do not know whether wearing the splint will be painful. When the splint is being fitted, too much pressure on the fingers and excessive tension at the possible wound site must be avoided. When the wound has healed and as pain thresholds permit, the splint can be remoulded as appropriate. Elastic Velcro is adhered dorsally to prevent the splint from twisting and turning, and is sufficiently wide to produce light pressure for elongating structures, if necessary. Participants are encouraged to contact the therapist for splint adjustments, if something prevents them from wearing it. Precautions will be specifically guided by patient-reported pain, as pain is indicative of possible micro tears in hand tissues, leading to scarring and more contractures. If the PIPJ contracture is severe (≥40 degrees) post-CCH, an even more-targeted splint for the specific joint will be constructed when the possible wound is sufficiently healed – a finger Gutter splint” (Figs. 6 and 7) or a three point finger splint (Figs. 8 and 9).

#### Exercises

Patients are instructed to perform the exercises at home several times a day for short sessions, completing 10 repetitions for each exercise.

**Fig. 10:**
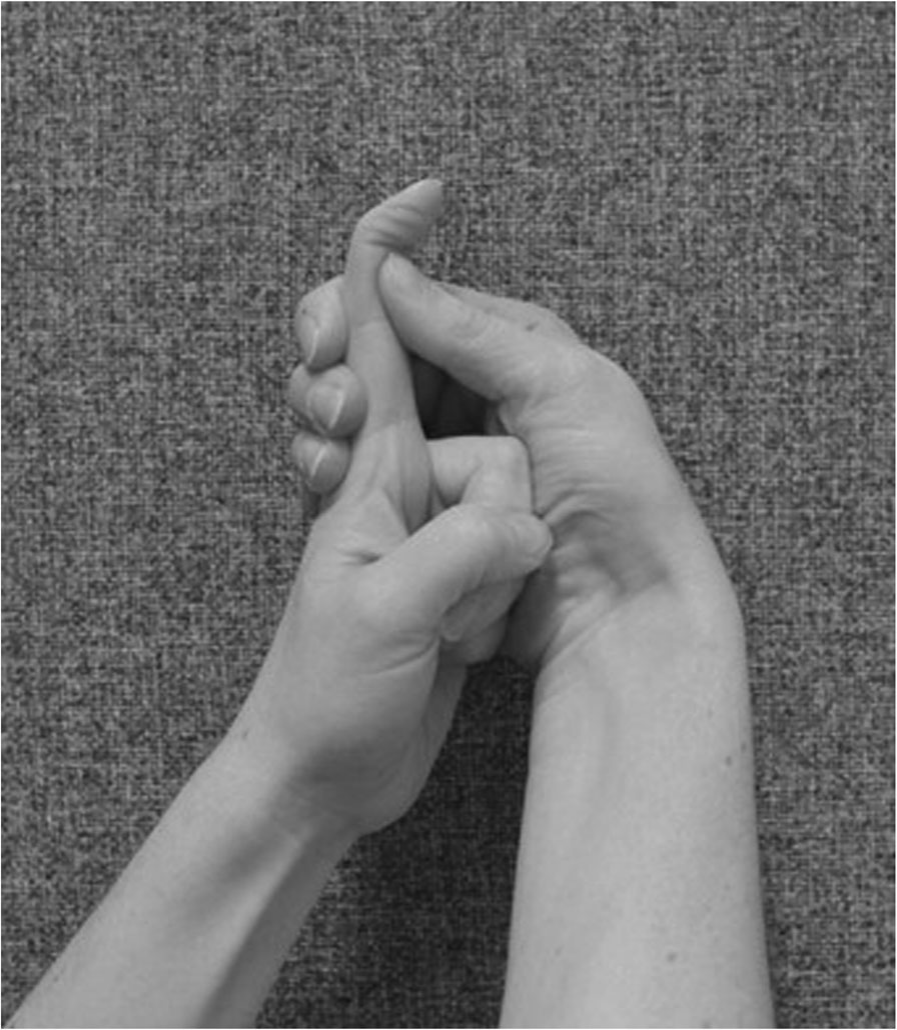
Isolated exercise for DIPJ

**Fig. 11:**
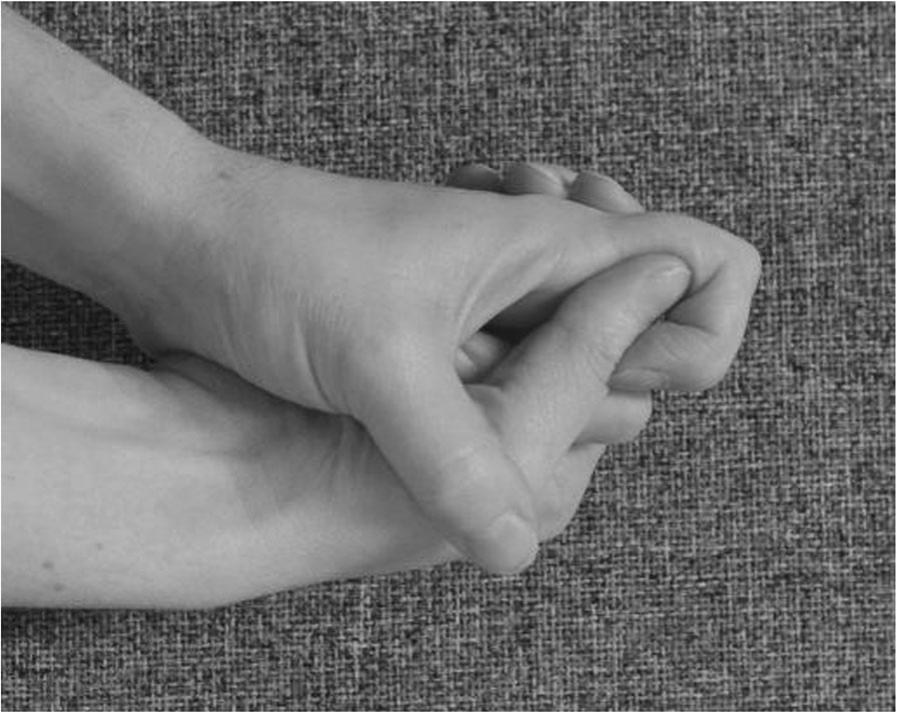
Isolated exercise for PIPJ

**Fig. 12:**
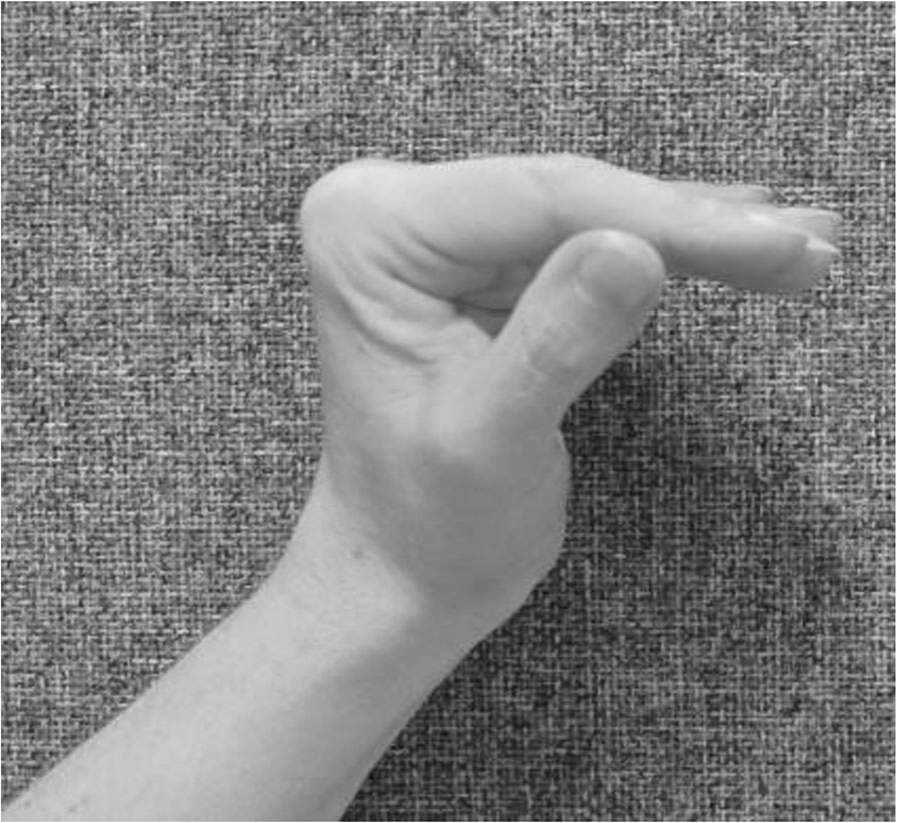
Isolated exercise for MCPJ

Hand exercises, if hand function is impaired:

- make a fist and stretch out the fingers to engage finger movement (Figs. 2 and 3)
- isolated exercises for the specific joint(s) (Fig. 10, 11 and 12)

**Fig. 13:**
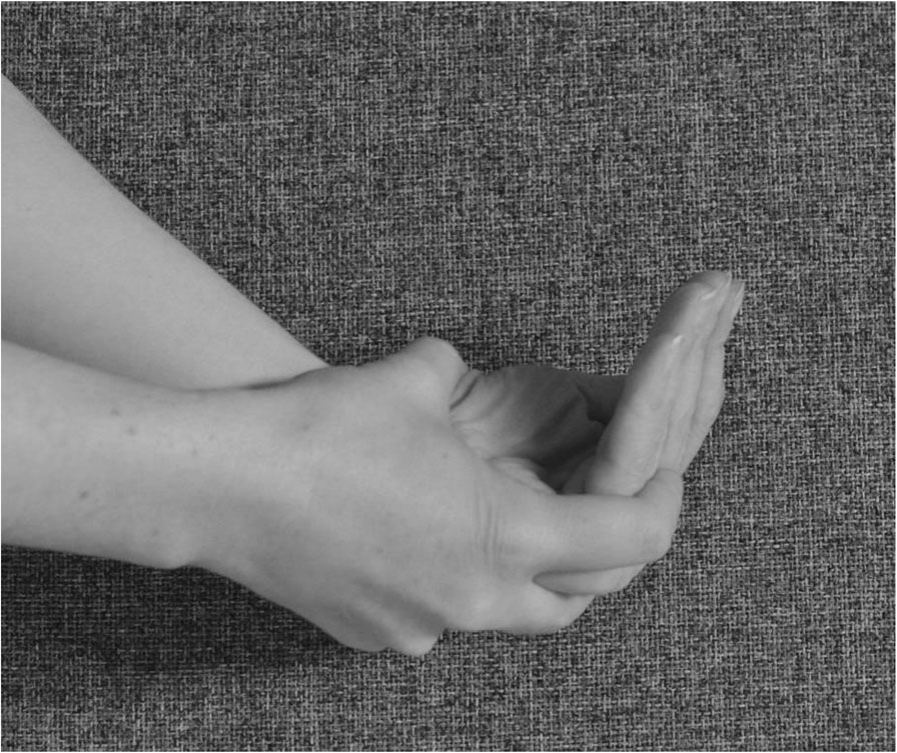
Extension of the PIPJ and DIPJ with the MCPJ blocked in flexion

Hand exercises, if the PIPJ is involved:

- flexion of the DIPJ with the PIPJ held in extension to lengthen the oblique retinacular ligaments (Fig. 10)
- extension of the PIPJ and DIPJ with the MCPJ blocked in flexion (Fig. 13)

#### Everyday activities

The therapy sessions will focus on activities listed in each patients’ COPM. The activities will be written in the diary to aid the patient’s memory. The therapist will guide the patients in how to use these activities as exercise, and will motivate the patients by explaining that performing rote exercises, in combination with other exercises through meaningful activities, produces better results [40].

### Treatment adherence

Patients in the intervention group will receive written instructions containing all the information about their treatment. They will meet the therapist individually as often as necessary, and for a minimum of three sessions: (1) at the day of the extension procedure; (2) within 4 weeks; and (3) at 6 weeks. They are free to contact the therapist if needed. This follow-up is designed to encourage treatment adherence.

### Outcome measures

Recommendations for assessing treatment progress for DC will be used [42, 43]. These include a combination of generic patient-reported outcome measures (PROM), a disease-specific questionnaire, a physical measure of active and passive individual joint ROM, and grip strength, using standardised protocols for DC assessment. An overview of the measures used is presented in Table 1 together with the schedule of when they are performed. Differences in performance parameters between the two groups will be evaluated. Subgroups will be similarly evaluated. Patient performance of and self-satisfaction with performance of everyday activities 1 year after CCH treatment will be the main outcome of interest.

**Table 1:** Schedule of enrolment, interventions, and assessments of patients with Dupuytren’s contracture

	Enrolment	Baseline tests	CCH treatment	6 weeks follow up	4 months follow up	1 year follow up
ENROLEMENT:						
Eligibility screen	X					
Informed consent	X					
Randomisation			X			
INTERVENTIONS:						
Hand therapy			X	X	X	
No hand therapy			X	X	X	X
ASSESSMENTS:						
Demographic and medical variables		X		X	X	X
Main outcome						
Everyday activities: COPM		X		X	X	X
Secondary outcomes						
Range of motion: Goniometer		X	X	X	X	X
Everyday living activities: URAM scale		X		X	X	X
Grip force: Jamar dynamometer		X		X	X	X
Pain: VAS		X		X	X	X
Cold hypersensitivity: Yes/no		X		X	X	X
Sick leave: Yes/no		X		X	X	X
Satisfaction with change in hand function: PGIC					X	X

### Primary outcome measure: Canadian model of occupational performance

The Canadian Occupational Performance Measure (COPM) is a client-centred tool, with which individuals identify and prioritise everyday issues that restrict or impact their performance of everyday living activities [44]. The Norwegian version of the COPM will be used [45]. The COPM is reliable [46], has good construct validity for DC [47], as well as criterion responsiveness [48]. It is a relevant instrument for the population of DC sufferers and captures performance problems that may be missed by other tests [47].

This self-perception of performance and satisfaction with performance is tracked over time by the tool. To optimise the relevance of the COPM in this study, participants are asked to identify activity limitations caused as a result of their DC. Participants rate the importance of each activity on a scale from 1 to 10, and they select a maximum of five activity limitations that they feel are the most important activities they want to improve. The participants evaluate each activity with regard to their actual performance and satisfaction with their performance on a scale from 1 to 10; higher values indicate better performance or greater satisfaction. The mean total performance score and satisfaction score will be calculated over the chosen activities. We deemed a change in score of 2 as a clinically important change, a value indicated in the literature for the COPM [44].

At each of the follow-up assessments, participants will rate their current performance and satisfaction with their performance of each of the five activities that they identified at baseline. Change scores are calculated using the previous scores and the current ones.

### Secondary outcomes

#### Unité Rhumatologique des affections de la Main (URAM) scale

To better compare the results of this proposed trial on DC with other studies, and to examine participants’ performance of predefined disease-specific activities, we will use the Norwegian version of the Unité Rhumatologique des Affections de la Main (URAM-N) scale [49]. Performance of nine predefined activity limitations is evaluated on six different degrees of performance. With the URAM-N, a maximum score of 45 is possible, indicating the worst possible condition. The original version of the URAM scale has strong convergence with the Tubiana scale (contractures of the joints) and self-assessed disability measured with a visual analogue scale (VAS) [3]. The clinimetric properties for the Norwegian scale have not been tested yet.

#### Range of motion (ROM)

In the majority of research on DC treatments, ROM is the primary outcome variable. In the present trial, group differences in mean change of ROM (active flexion and active/passive extension of each treated joint) will be evaluated. DC recurrence rates will also be evaluated in the four groups. We define a successful result as (1) a joint that achieves an extension to 0–5 degrees of full extension, and (2) a clinical improvement of more than 50% reduction of the original contracture [12]. We adopted recommendations to improve ROM measuring and reporting [39, 50]. A Rolyan goniometer with a precision of five-degree intervals (range, 0–180 degrees) will be used to measure ROM, as it is the established ROM-measuring tool routinely used in a clinical context. Only treated joints will be measured for ROM.

The Swedish Hand Surgical Quality National Register (HAKIR) reached a consensus on how to measure ROM with a goniometer [51]. This method will be followed, and in addition, we will measure with the elbow resting on a table, hand up, and wrist in neutral position. MCPJs will be both flexed and then extended when measuring PIPJ extension. Active flexion and both passive and active extension of each treated joint will be measured separately. The results will be tabulated, as recommended by Kan et al. [39]. Extension deficit is marked with a minus sign, full extension and hyperextension is defined as zero degrees. We define DC recurrence as an increase in treated-joint contracture at the one-year follow-up of at least 20 degrees compared to the six-weeks follow-up measurement [39].

Following the COSMIN criteria [52], one systematic review found a limited level of evidence for an acceptable reliability in the dorsal measurement method of goniometry assessment of the finger joints, and an unknown level of evidence for the measurement error [53].

#### Grip force

We will evaluate group differences in mean change scores on grip force. A calibrated Jamar dynamometer will be used, which is a hydraulic hand-held tool capable of measuring grip force from 0 to 90 kg. A peak hold needle retains the highest reading until the device is reset. The Swedish HAKIR manual for measuring grip force will be followed [51]. The measurement shows good test-retest, inter-tester, and intra-tester reliability [54–57].

#### Pain

For characteristics that can take on values spanning a continuum that cannot be easily measured directly, a reliable proxy measure can be obtained with a VAS ranging from 0 to 10 cm. Participants are asked to indicate the intensity of their pain symptom along a straight line measuring 10 cm. The end points of the line are labelled as the extreme lower and upper limits of the describable pain, with 10 indicating the worst possible, and 1 indicating no pain [58]. We will ask the participants how much pain they experienced in the treated hand in the last 24 h before the present assessment. The VAS which the participant is asked to mark has only the endpoints labelled with no numbers on it. On the backside of the VAS there will be numbers 0-100 mm, for the tester to register.

#### Patient global impression of change

At the four-month and one-year follow-ups, participants will be asked how satisfied or unsatisfied they are with the change in hand function compared to the situation prior to CCH treatment. The Patient Global Impression of Change (PGIC) will be used [59]. The questions for the PGIC refer to the following statement: “My hand function has changed since the collagenase treatment.” “Are you satisfied with the change?” The patients are instructed to state how satisfied or dissatisfied they are across 7 levels of satisfaction ranging from not satisfied at all to very satisfied [59].

#### Additional questions

Basic demographic information will be collected. Indications for treatment in other fingers in the same hand or in the other hand will be recorded at baseline and re-assessed at each follow-up. We will ask the participants whether they are sensitive to cold and about sick leave, both at baseline and at the follow-ups. Difficulties with oedema and scarring will be queried at the follow-ups. The participants in the intervention group will record in a diary if they have a wound, scar, or swelling; whether they used the splint and how much; and whether they used the COPM activities as exercise. The diary will be retrieved by a secretary at the four-month follow-up and the data analyst will analyse these data after the final follow-up at 1 year.

### Data management

All data collected will be entered continuously, as they become available, into SPSS (IBM SPSS Statistics for Windows, Version 22.0. Released 2013. Armonk, NY: IBM Corp) for subsequent analysis. The data will be maintained on a secure HUH research server, secured in a research database, with access granted only to the primary investigators. Identification codes of the participants and their group allocation will be kept apart from the outcomes and demographics data. Published data will be anonymised.

### Statistical analysis

We determined the appropriate sample size according to the minimal clinically important change for the COPM [44]. COPM gives two main outcomes; performance of activity, and satisfaction with performance. There is strong correlation between the two [60], and we therefore used both for the calculation of the sample size. Van de Ven-Stevens reported a standard deviation (SD) of 1.1 for performance and 0.8 for satisfaction for DC patients assessed with the COPM [47]. For this trial, we assumed a more conservative value, setting the SD to 2.0. For our power analysis, we conservatively assumed we would detect an effect only in the PIPJ group, which would produce a mean COPM difference of 1.0 between the hand therapy and control groups. Power analysis shows that we require 64 participants in each group to detect this difference for a two-sided t-test, with a significance level of *p* < 0.05 and power of 0.8. These parameters suggest we need 32 patients in each PIPJ and MCPJ subgroup. This would enable us to detect a mean difference of 2.0 (SD = 2) between PIPJ and MCPJ in the intervention group, with a power of 0.98. Assuming 20% loss-to-follow-up [22], we require 80 patients in the no hand therapy group and 80 in the hand therapy group.

Descriptive statistics will be used to characterise the participants’ data at baseline and follow-ups, and separately for the data of those participants who might drop out from the study. The baseline values of every measure will also be used to describe the randomised groups to determine their comparability.

The effect of post-CCH hand therapy at 1 year will be assessed using analysis of covariance (ANCOVA). The development of changes over time will be evaluated using a linear mixed-effects model, with time, intervention group, and their interaction as predictors. An intention-to-treat analysis will be used. The predictive value of PIPJ/MCPJ will be assessed by adding the interaction between group and PIPJ/MCPJ to the models. Results will be summarised by graphical illustrations.

The significance level is set to *p* < 0.05. SPSS and Matlab® (MATLAB and Statistics Toolbox Release 2012b, The MathWorks, Inc., Natick, MA, USA) will be used for statistical analyses and creation of Figures.

## Discussion of methodological limitations and strengths

All clinical trials require completely transparent protocols [61]. By using the SPIRIT recommendations for minimum relevant protocol items, we achieved this goal of transparency. This paper describes the study design for an RCT investigating the effects of hand therapy, or lack of it, on patients who have undergone CCH treatment for DC. The study is also designed to determine whether differences exist in the outcome measures of patients with DC of the PIPJ involved or of only the MCPJ. The design is not optimal, as patients and therapists cannot be blinded to the treatment modality. This is a common problem in clinical studies. However, the therapist assessing the participants’ performance and satisfaction, the surgeon, and the data analyst will be blinded to the treatment modality.

In drafting our research plan, we considered recommendations in the empirical literature on DC. It is impossible to fully control some factors or issues. For example, the presentation of DC varies considerably, making each DC case unique. Thus, patients will respond differently to the CCH treatment and will thus require different therapeutic interventions to address their unique complaints. Also, as DC is a chronic disorder, the disease itself remains active even after treatment and thus contractures may continue to occur. Another issue that we cannot control is the structural status or integrity of tissues surrounding the affected joint. Those structures may be so stiff that it would be impossible to fully straighten the affected joint. These issues make research on DC treatments and rehabilitation challenging. Our study, however, has attempted to control for some of these variations by incorporating two subgroups, one comprising patients with PIPJ contractures and the other comprising patients with only MCPJ contractures. We surmise that one of the subgroup of patients may require more intensive hand therapy than the other.

A potential source of limitations is our choice of outcome measures. One of these measures is the URAM scale, a disease-specific self-assessment scale that measures disability due to DC [20]. The URAM scale has been criticised for not embracing all of the most important activity limitations of DC patients [20]. Thus, its face validity might need to be reassessed and perhaps modified. Another tool we use in this RCT is COPM, a tool that identifies occupational activities patients have difficulty performing [47]. The COPM may reveal important activity limitations not identified by other patient-related outcome measures [47], as it can be used to gather information about the most important activities patients perform in daily life. COPM will enable us to determine and gauge the patients’ satisfaction with their activity performance. This may give healthcare professionals wider insight into the patients’ activity limitations after CCH treatment. One reason it may not appear to assess certain activity limitations is that our experience shows that patients may actually experience disease-related problems, but they do not recognise them as such and thus do not note them accordingly on the COPM. Using two complementary assessment scales-COPM and URAM, ensures that all activities salient to DC patients are assessed.

One possible limitation with the study relates to DC nomenclature and terminology. Studying contracture recurrence and comparing recurrent rates from the literature is a challenge, since these terms were not uniformly defined until 2017 [39]. Different definitions for recurrence greatly influence the reported recurrence rates. In addition, ROM lacks clarity in earlier research and there are different methods for measuring it [50, 53]. In the context of a consensus definition of DC recurrence, it is recommended to measure each joint separately [39]. HAKIR describes a consensus protocol on how to measure the ROM [51].

There are other various factors that can affect our study outcomes, such as to what extent patients perform daily activities and whether they employ other strategies to improve their hand function. To date, no studies have been published that examined how performing daily activities can improve hand function in DC patients. In the present study, the hand therapy group will be encouraged by the occupational therapist to engage in daily activities. The therapist will demonstrate how these activities can be used to improve their hand mobility and function, and discuss its benefits. The control group will also be using their hands for daily activities and they may improve. We will determine whether this type of instruction will improve the performance of everyday activities compared to if not, wherein patients do not undergo hand therapy and thus do not receive specific instructions on exercising their hand as they perform daily activities. Through regular study follow-ups, the no hand therapy group will become aware of what functions we expect to improve. If the control patients did not participate in our study, they would be unaware of what functions to improve, and thus might not try to improve them. We cannot control what the patients do to improve their hand function on their own.

The hypotheses are about the patient performance and satisfaction with performance of everyday activities, and about the anticipation that the joint involvement has an impact. The study will look at therapy as a whole package; it will not be possible to tell which part of the therapy is beneficial or not. The question if a splint is needed will not be answered, and have to be investigated in a later study if therapy show beneficial effect.

Research on DC and its treatment is challenging, both in regard to the kind of medical approach used and to the kind of therapy selected after treatment. Our goal in this study is to produce empirically defined recommendations for therapy following CCH treatment, taking into consideration that each patient is unique, and that recurrence can occur because the disease is not curable. The intervention will differ slightly according to the patients’ symptoms, reflecting standard clinical practice. The study will objectively answer the question of whether or not hand therapy makes a difference on activity performance and patient self-satisfaction with performance, and whether the particular joints involved (i.e., PIPJ or MCPJ) differ with respect to performance and self-satisfaction outcomes. These results will provide an objective basis to inform policies of referral for hand therapy after CCH treatment.

## Data Availability

Datasets used and/or analysed during the current study will be made available from the corresponding author on reasonable request. Data on which the conclusions of the paper rely will be published in the manuscript.

## Abbreviations

ANCOVA: Analysis of covariance
CCH: Collagenase Clostridium Histolyticum
COPM: Canadian Occupational Performance Measure
CRPS: Complex regional pain syndrome
DC: Dupuytren’s contracture
DIPJ: Distal interphalangeal joint
HAKIR: Swedish Hand Surgical Quality National Register
HT: Hand therapy
HUH: Haukeland University Hospital
MCPJ: Metacarpophalangeal joint
PGIC: Patient Global Impression of Change
PIPJ: Proximal interphalangeal joint
PROM: Patient-reported outcome measures
REC: Regional ethical committee
ROM: Range of motion
SD: Standard deviation
SPIRIT: Standard protocol items: recommendations for interventional trials
URAM: Unité Rhumatologique des Affections de la Main
URAM-N: Unité Rhumatologique des Affections de la Main Norwegian version
VAS: Visual analogue scale

## References

[1] Hindocha S, McGrouther DA, Bayat A (2009). Epidemiological evaluation of Dupuytren's disease incidence and prevalence rates in relation to etiology. Hand..

[2] Black EM, Blazar PE (2011). Dupuytren disease: an evolving understanding of an age-old disease. The Journal of the American Academy of Orthopaedic Surgeons.

[3] Bernabe B, Lasbleiz S, Gerber RA, Cappelleri JC, Yelnik A, Orcel P (2014). URAM scale for functional assessment in Dupuytren's disease: a comparative study of its properties. Joint, bone, spine : revue du rhumatisme.

[4] Nordenskjold J, Englund M, Zhou C, Atroshi I (2017). Prevalence and incidence of doctor-diagnosed Dupuytren's disease: a population-based study. J Hand Surg-Eur Vol.

[5] Wilburn J, McKenna SP, Perry-Hinsley D, Bayat A (2013). The impact of Dupuytren disease on patient activity and quality of life. The Journal of hand surgery.

[6] Kan HJ, de Bekker-Grob EW, van Marion ES, van Oijen GW, van Nieuwenhoven CA, Zhou C (2016). Patients' preferences for treatment for Dupuytren's disease: a discrete choice experiment. Plast Reconstr Surg.

[7] Engstrand C, Boren L, Liedberg GM (2009). Evaluation of activity limitation and digital extension in Dupuytren's contracture three months after fasciectomy and hand therapy interventions. Journal of hand therapy: official journal of the American Society of Hand Therapists..

[8] Akhavani MA, McMurtrie A, Webb M, Muir L (2015). A review of the classification of Dupuytren's disease. J Hand Surg Eur Vol.

[9] Wilbrand S, Warwick D (2015). Collagenase. Dupuytren's disease, FESSH instructional course 2015.

[10] Manning CJ, Delaney R, Hayton MJ (2014). Efficacy and tolerability of day 2 manipulation and local anaesthesia after collagenase injection in patients with Dupuytren's contracture. J Hand Surg Eur Vol.

[11] Peimer CA, Blazar P, Coleman S, Kaplan FT, Smith T, Lindau T (2015). Dupuytren contracture recurrence following treatment with collagenase Clostridium histolyticum (CORDLESS [collagenase option for reduction of Dupuytren long-term evaluation of safety study]): 5-year data. The Journal of hand surgery..

[12] Hurst LC, Badalamente MA, Hentz VR, Hotchkiss RN, Kaplan FT, Meals RA (2009). Injectable collagenase clostridium histolyticum for Dupuytren's contracture. N Engl J Med.

[13] Fairplay T, Larsen D, Jerosch-Herold C (2015). The role of hand rehabilitation after surgical and "non-surgical" procedures for Dupuytren's disease. In: warwick D, editor. Dupuytren's disease, FESSH Instructional Course 2015.

[14] Krefter C, Marks M, Hensler S, Herren DB, Calcagni M (2017). Complications after treating Dupuytren's disease. A systematic literature review. Hand surgery & rehabilitation.

[15] Sanjuan-Cervero R, Carrera-Hueso FJ, Vazquez-Ferreiro P, Gomez-Herrero D (2017). Adverse effects of collagenase in the treatment of Dupuytren disease: a systematic review. BioDrugs : clinical immunotherapeutics, biopharmaceuticals and gene therapy.

[16] Fagernaes C, Mallet S. The risk of skin tear in Dupuytren's disease when treated with collagenase. Dan Med J. 2018;65(5). 29726315

[17] Sanjuan-Cervero R, Carrera-Hueso FJ, Oliver-Mengual S, Ramon-Barrios MA, Peimer CA, Fikri-Benbrahim N (2018). Skin laceration in collagenase Clostridium histolyticum treatment for Dupuytren's contracture. Orthop Nurs.

[18] Bradley J, Warwick D (2016). Patient satisfaction with collagenase. The Journal of hand surgery..

[19] Kan HJ, Verrijp FW, Huisstede BM, Hovius SE, van Nieuwenhoven CA, Selles RW (2013). The consequences of different definitions for recurrence of Dupuytren's disease. Journal of plastic, reconstructive & aesthetic surgery: JPRAS.

[20] Rodrigues JN, Zhang W, Scammell BE, Davis TR (2015). What patients want from the treatment of Dupuytren's disease--is the unite Rhumatologique des affections de la Main (URAM) scale relevant? The journal of hand surgery. European volume.

[21] Huisstede BM, Gladdines S, Randsdorp MS, Koes BW. Effectiveness of conservative, surgical, and post-surgical interventions for Trigger finger, Dupuytren's disease, and De Quervain's disease. A systematic review. Archives of physical medicine and rehabilitation. 2017. 10.1016/j.apmr.2017.07.014 28860097

[22] Jerosch-Herold C, Shepstone L, Chojnowski AJ, Larson D, Barrett E, Vaughan SP (2011). Night-time splinting after fasciectomy or dermo-fasciectomy for Dupuytren's contracture: a pragmatic, multi-Centre, randomised controlled trial. BMC Musculoskelet Disord.

[23] Arno AI, Gauglitz GG, Barret JP, Jeschke MG (2014). Up-to-date approach to manage keloids and hypertrophic scars: a useful guide. Burns : journal of the International Society for Burn Injuries.

[24] Miller LK, Jerosch-Herold C, Shepstone L (2017). Clinical assessment of hand oedema: a systematic review. Hand Ther.

[25] Miller LK, Jerosch-Herold C, Shepstone L (2017). Effectiveness of edema management techniques for subacute hand edema: a systematic review. Journal of hand therapy : official journal of the American Society of Hand Therapists..

[26] Reno F, Sabbatini M, Lombardi F, Stella M, Pezzuto C, Magliacani G (2003). In vitro mechanical compression induces apoptosis and regulates cytokines release in hypertrophic scars. Wound repair and regeneration: official publication of the wound healing society [and] the European tissue repair. Society..

[27] Kim JS, Hong JP, Choi JW, Seo DK, Lee ES, Lee HS (2016). The efficacy of a silicone sheet in postoperative scar management. Adv Skin Wound Care.

[28] Degreef Ilse, Brauns Annelien (2016). Splinting as a Therapeutic Option in Dupuytren Contractures. Dupuytren Disease and Related Diseases - The Cutting Edge.

[29] Skirven TM, Bachoura A, Jacoby SM, Culp RW, Osterman AL (2013). The effect of a therapy protocol for increasing correction of severely contracted proximal interphalangeal joints caused by dupuytren disease and treated with collagenase injection. The Journal of hand surgery..

[30] Arora R, Kaiser P, Kastenberger TJ, Schmiedle G, Erhart S, Gabl M (2016). Injectable collagenase Clostridium histolyticum as a nonsurgical treatment for Dupuytren's disease. Operative Orthopadie und Traumatologie.

[31] Samargandi OA, Alyouha S, Larouche P, Corkum JP, Kemler MA, Tang DT (2017). Night Orthosis After Surgical Correction of Dupuytren Contractures: A Systematic Review. The Journal of hand surgery.

[32] Goldsmith N, Juzl E, Mee S. Management of the PIP joint. BAHT level II; St. Georges Hospital, London 7–9 september: NES Hand Therapy training; 2016.

[33] Colditz J. Therapist's management of the stiff hand. In: Skirven TMO, A.L. Fedorczyk, J. Amadio P.C., editor. Rehabilitation of the hand and upper extremity. 1. 6 ed. Philadelphia: Mosby Elsevier; 2011. p. 894–922.

[34] Harvey LA, Katalinic OM, Herbert RD, Moseley AM, Lannin NA, Schurr K (2017). Stretch for the treatment and prevention of contracture: an abridged republication of a Cochrane systematic review. J Phys.

[35] Roll SC, Hardison ME (2017). Effectiveness of Occupational Therapy Interventions for Adults With Musculoskeletal Conditions of the Forearm, Wrist, and Hand: A Systematic Review. The American journal of occupational therapy: official publication of the American Occupational Therapy Association.

[36] Che Daud AZ, Yau MK, Barnett F, Judd J, Jones RE, Muhammad Nawawi RF (2016). Integration of occupation based intervention in hand injury rehabilitation: a randomized controlled trial. Journal of hand therapy : official journal of the American Society of Hand Therapists..

[37] Rostami HR, Akbarfahimi M, Hassani Mehraban A, Akbarinia AR, Samani S (2017). Occupation-based intervention versus rote exercise in modified constraint-induced movement therapy for patients with median and ulnar nerve injuries: a randomized controlled trial. Clin Rehabil.

[38] Guzelkucuk U, Duman I, Taskaynatan MA, Dincer K (2007). Comparison of therapeutic activities with therapeutic exercises in the rehabilitation of young adult patients with hand injuries. The Journal of hand surgery..

[39] Kan HJ, Verrijp FW, Hovius SER, van Nieuwenhoven CA, Dupuytren Delphi G, Selles RW (2017). Recurrence of Dupuytren's contracture: a consensus-based definition. PLoS One.

[40] Harden RN, Bruehl S, Perez RS, Birklein F, Marinus J, Maihofner C (2010). Validation of proposed diagnostic criteria (the "Budapest criteria") for complex regional pain syndrome. Pain..

[41] Al-Shaqsi S, Al-Bulushi T (2016). Cutaneous scar prevention and management: overview of current therapies. Sultan Qaboos Univ Med J.

[42] Ball C, Pratt AL, Nanchahal J (2013). Optimal functional outcome measures for assessing treatment for Dupuytren's disease: a systematic review and recommendations for future practice. BMC Musculoskelet Disord.

[43] van de Ven-Stevens LA, Graff MJ, Selles RW, Schreuders TA, van der Linde H, Spauwen PH (2015). Instruments for assessment of impairments and activity limitations in patients with hand conditions: a European Delphi study. J Rehabil Med.

[44] Law M, Baptiste S, Carswell A, McColl MA, Polatajki H, Pollock N. COPM the Canadian occupational performance measure. 5 ed. Canada: CAOT publications ACE; 2014.

[45] Kjeken I, Slatkowsky-Christensen B, Kvien TK, Uhlig T (2004). Norwegian version of the Canadian occupational performance measure in patients with hand osteoarthritis: validity, responsiveness, and feasibility. Arthritis Rheum.

[46] Kjeken I, Dagfinrud H, Uhlig T, Mowinckel P, Kvien TK, Finset A (2005). Reliability of the Canadian occupational performance measure in patients with ankylosing spondylitis. J Rheumatol.

[47] van de Ven-Stevens LA, Graff MJ, Peters MA, van der Linde H, Geurts AC (2015). Construct validity of the Canadian occupational performance measure in participants with tendon injury and Dupuytren disease. Phys Ther.

[48] Eyssen IC, Steultjens MP, Oud TA, Bolt EM, Maasdam A, Dekker J (2011). Responsiveness of the Canadian occupational performance measure. J Rehabil Res Dev.

[49] Beaudreuil J, Allard A, Zerkak D, Gerber RA, Cappelleri JC, Quintero N (2011). Unite Rhumatologique des affections de la Main (URAM) scale: development and validation of a tool to assess Dupuytren's disease-specific disability. Arthritis care & research.

[50] Pratt AL, Ball C (2016). What are we measuring?. A critique of range of motion methods currently in use for Dupuytren's disease and recommendations for practice BMC musculoskeletal disorders.

[51] Rehabilitation clinics in Göteborg L, Malmö, Stockholm, Umeå, Uppsala and Örebro. Nationell manual för mätning av rörelse och styrka. Sweeden: Handsurgery quality register; 2016.

[52] Mokkink LB, Terwee CB, Patrick DL, Alonso J, Stratford PW, Knol DL (2010). The COSMIN study reached international consensus on taxonomy, terminology, and definitions of measurement properties for health-related patient-reported outcomes. J Clin Epidemiol.

[53] van Kooij YE, Fink A (2017). Nijhuis-van der Sanden MW, Speksnijder CM. The reliability and measurement error of protractor-based goniometry of the fingers: a systematic review. Journal of hand therapy : official journal of the American Society of Hand Therapists.

[54] Mathiowetz V, Weber K, Volland G, Kashman N (1984). Reliability and validity of grip and pinch strength evaluations. The Journal of hand surgery..

[55] Peolsson A, Hedlund R, Oberg B (2001). Intra- and inter-tester reliability and reference values for hand strength. J Rehabil Med.

[56] Lindstrom-Hazel D, Kratt A, Bix L (2009). Interrater reliability of students using hand and pinch dynamometers. The American journal of occupational therapy : official publication of the American Occupational Therapy Association.

[57] Roberts HC, Denison HJ, Martin HJ, Patel HP, Syddall H, Cooper C (2011). A review of the measurement of grip strength in clinical and epidemiological studies: towards a standardised approach. Age Ageing.

[58] Polit DF, Beck CT, editors. Nursing research: Generating and assessing evidence for nursing practice. 8 ed: Lippincott Williams & Wilkins; 2008.

[59] Farrar JT, Young JP, LaMoreaux L, Werth JL, Poole RM (2001). Clinical importance of changes in chronic pain intensity measured on an 11-point numerical pain rating scale. Pain..

[60] Padankatti SM, Macaden AS, Cherian SM, Thirumugam M, Pazani D, Kalaiselvan M (2011). A patient-prioritized ability assessment in haemophilia: the Canadian occupational performance measure. Haemophilia..

[61] Chan AW, Tetzlaff JM, Gotzsche PC, Altman DG, Mann H, Berlin JA (2013). SPIRIT 2013 explanation and elaboration: guidance for protocols of clinical trials. Bmj..

